# Use of an Innovative Web-Based Laboratory Surveillance Platform to Analyze Mixed Infections Between Human Metapneumovirus (hMPV) and Other Respiratory Viruses Circulating in Alberta (AB), Canada (2009–2012)

**DOI:** 10.3390/v4112754

**Published:** 2012-11-05

**Authors:** Sumana Fathima, Bonita E. Lee, Jennifer May-Hadford, Shamir Mukhi, Steven J. Drews

**Affiliations:** 1 Provincial Laboratory for Public Health (ProvLab), 3030 Hospital Dr. NW, Calgary, Alberta, T2N 4W4, Canada; Email: sumana.fathima@albertahealthservices.ca; 2 University of Alberta, Room 3-588B, ECHA, 11405 – 87 Avenue, Edmonton, Alberta, Canada T6G 1C9; Email: bonita.lee@albertahealthservices.ca; 3 Public Health Agency of Canada, 130 Colonnade Road A.L. 6501H Ottawa, Ontario, K1A 0K9, Canada; Email: jennifer.may-hadford@phac-aspc.gc.ca; 4 Canadian Network for Public Health Intelligence, Public Health Agency of Canada, 1015 Arlington St, Winnipeg, Manitoba, R3E 3R2, Canada; Email: shamir.nizar.mukhi@phac-aspc.gc.ca; 5 University of Calgary, 2500 University Drive Northwest, Calgary, Alberta, T2N 1N4, Canada; Email: steven.drews@albertahealthservices.ca

**Keywords:** hMPV, co-infection, testing, epidemiology, respiratory

## Abstract

We investigated the proportions of mono *vs*. mixed infections for human metapneumovirus (hMPV) as compared to adenovirus (ADV), four types of coronavirus (CRV), parainfluenza virus (PIV), RSV, and enterovirus/rhinovirus (ERV) in Alberta, Canada. Using the Data Integration for Alberta Laboratories (DIAL) platform, 26,226 respiratory specimens at ProvLab between 1 July 2009 and 30 June 2012 were selected and included in the study. Using the Respiratory Virus Panel these specimens tested positive for one or more respiratory virus and negative for influenza A and B. From our subset hMPV was the fourth most common virus (n=2,561) with 373 (15%) identified as mixed infection using DIAL. Mixed infection with hMPV was most commonly found in infants less than 6 months old and ERV was most commonly found in mixed infection with hMPV (230/373, 56%) across all age groups. The proportion of mixed-infection *vs*. mono-infection was highest for ADV (46%), followed by CRV 229E (32%), CRV HKU1 (31%), CRV NL63 (28%), CRV OC43 (23%), PIV (20%), RSV (17%), hMPV (15%) and ERV (13%). hMPV was significantly more likely to be identified in mono infection as compared with ADV, CRV, PIV, and RSV with the exception of ERV [p<0.05].

## 1. Introduction

Respiratory tract infections are a global public health concern and in Canada is the eighth leading cause of death in 2009 [1]. Human metapneumovirus (hMPV) is an RNA virus belonging to *Paramyxoviridae *family. The virus was identified by researchers in the Netherlands in 2001 as an important cause of respiratory infections that affect all age groups [2]. A study from Saskatchewan, Canada by Liu *et al.* [3], using ELISA showed that seroprevalence for hMPV approaches 99% by young adulthood. This virus can affect any age group but several studies have shown that hMPV is a leading cause in lower respiratory tract infections in children [4,5,6] but it also affects the elderly [7]. Clinical manifestations are similar to Respiratory Syncytial Virus (RSV) primarily leading to pneumonia and bronchiolitis [8]. In Canada, outbreaks associated with hMPV have been reported in Alberta, British Columbia and Quebec mainly in long term and senior care facilities [9,10,11]. Other common respiratory viruses in these settings include influenza A (FLUA), influenza B (FLUB), parainfluenza virus (PIV), enterovirus/rhinovirus (ERV), adenovirus (ADV), and coronavirus (CRV), which all can cause lower respiratory tract infections [12,13].

Provincial Laboratory for Public Health (ProvLab) in Alberta provides testing for all respiratory virus pathogens for the province of Alberta and surrounding Northern Territories (excluding Yukon) of Canada. The Diagnostic testing algorithm for respiratory virus at ProvLab changed during the 2009 H1N1 pandemic. From April 2009, respiratory specimens arriving at ProvLab were screened by an in-house real-time reverse-transcriptase (RT)-PCR for influenza A [14]. Specimens that were positive for influenza A were also subtyped for seasonal H3, seasonal H1 and pandemic H1N1 (2009) genes by RT-PCR. A cost effective approach was adopted in June 2009, to only test specimens negative for both influenza A and B by either singleplex or multiplex real time PCR assays using the Respiratory Virus Panel (RVP) classic assay, a multiplexed assay which detects multiple respiratory viral pathogens including FLUA, FLUB, PIV, ERV, ADV, 4 types of CRV, RSV, and hMPV [15]. Exceptions to this testing policy include samples submitted from a provincial influenza-like-illness surveillance program (Tarrant Viral Watch) and some samples from patients with severe illness and admission to the intensive care units.

In Alberta, a unique platform was developed for laboratory-based surveillance called Data Integration of Alberta Laboratories (DIAL). DIAL is a secure web based platform, which is used to extract, interpret, collate and analyze respiratory virus testing data from ProvLab, Laboratory Information System (LIS) in real time [16]. DIAL has an automatic engine that extracts raw specimen-based laboratory data from ProvLab LIS, including patient demographics, information of physician and submitting agencies, and test data for specific targets. The second and most important component of DIAL is a built-in Automated Interpretation Engine (AIE) which provides clinically relevant interpretation and final target-specific classifications for each specimen. Finally, DIAL also has an analytical engine which allows users to select and create specific data sets for various targets by different factors, e.g., patient demographics, geographic distribution, time periods, testing methods and perform different types of analysis including graphical presentations, tables, maps, rate calculation and trending analysis. In the case of respiratory specimens from ProvLab*,* DIAL’s AIE was designed to assign positive and negative classifications for each respiratory virus as well as a summary classification that classifies each specimen as: 1) only positive for one specific virus, 2) mixed infection with more than one respiratory virus or 3) negative for all respiratory viruses. Using these final classifications, positive and negative specimens for each virus can easily be selected in DIAL for further analysis.

In this study we used DIAL to select specimen-based data and investigated the proportions of mono *vs* mixed infections for hMPV as compared to ADV, CRV, ERV, PIV and RSV for a period of three years, 1 July 2009 to 30 June 2012. In order to create a uniform dataset for this study, we excluded all samples that tested positive for influenza A or B by the in-house real-time PCR assays and included only samples that had undergone RVP testing.

## 2. Results and Discussion

Using DIAL, 36,824 specimens were identified as positive for one or more respiratory virus during the study period. A total of 10,598 were excluded from this study with 9,340 tested positive for influenza A, 1,065 for influenza B, 7 for both influenza A and B and 185 specimens not tested by RVP even though they were negative for influenza. For the 26,226 RVP positive specimens included in the study, 10,042 (38%) were received between July 2009 and June 2010, 8,450 (32%) between July 2010 and June 2011 and 7,734 (30%) between July 2011 and June 2012. Mixed infection (having more than one virus identified) was found in 2,330 (9%) specimens and 23,896 (91%) had mono-infection (having only one virus identified). The majority of the specimens were collected from the upper respiratory tract as nasopharyngeal/nasal/throat swabs or nasopharyngeal aspirates 84% (n=22,013), 12% (n=3076) as respiratory samples with unspecified source, 4% (n=917) were from the lower respiratory tract e.g., endotracheal aspirates or bronchaveolar lavage, and remaining as unknown sample types and a few tissues and sterile body fluid.

The age distribution of specimens submitted and tested positive for hMPV is summarized in Table 1. The highest number of specimens received was from patients less than 6 months old and the proportion of specimens tested positive for hMPV ranged from 4–19% among the different age groups (p<0.001, Chi Square test). Overall, mixed infection was detected in 15% of specimens tested positive for hMPV. Using specimens from the youngest age group (less than six months old) as the reference age group, there was significant difference for the proportion of mixed hMPV infection among the various age groups (p<0.05, Binary Logistic Regression) (Table 1). Mixed infection with hMPV was most commonly found in specimens from patients younger than six months and rarely in specimens submitted from older than 70 years old.

Among the 373 specimens with mixed infections with hMPV, the three most commonly found virus was ERV, RSV and PIV, which also were the three most common viruses detected in all the specimens (Table 2). In comparison with ADV, four types of CRV, PIV, and RSV, hMPV had a significantly lower proportion of mixed infection specimens [χ^2 ^with Bonferroni’s correction, p=<0.05] and there was no significant difference of mixed infection comparing hMPV and ERV [χ^2 ^with Bonferroni’s correction, p=0.06].

The age distribution of virus found in mixed infection with hMPV is summarized in Table 3. ERV was the most commonly found virus in hMPV mixed infection across all age groups.

**Figure 1:**
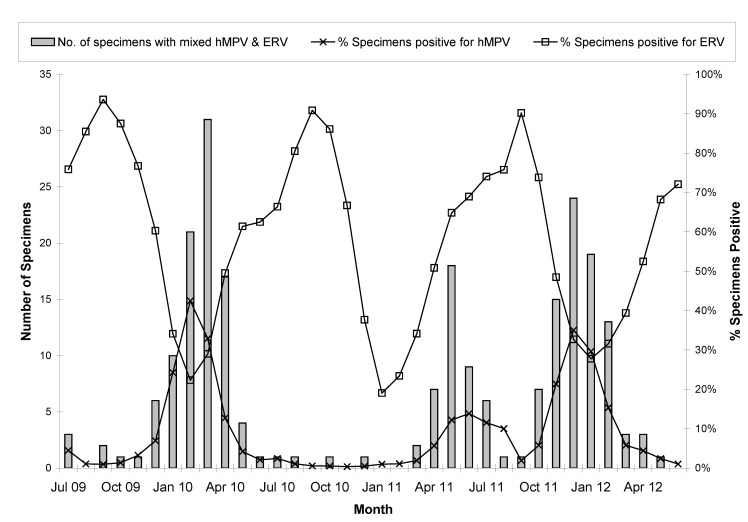
Monthly distribution of hMPV and ERV along with mixed infection with these two viruses.

Peak hMPV activity was observed in February 2010, June 2011 and November 2011, whereas peak ERV activity was found in the month of September in three consecutive years (2009, 2010 and 2011) (Figure 1). Specimens with mixed infection with these two viruses followed the trend and circulatory pattern of hMPV.

**Table 1:** Age distribution of specimens tested positive for metapneumovirus (hMPV) using Respiratory Virus Panel (RVP) and the number and % of mixed infection with hMPV.

Age groups	Number of specimens tested	Number of specimens tested positive for hMPV	Number of specimens with mixed hMPV infection	% with Mixed infection
	(n=26,226)	(%) (n=2,561)	(n=373)	
Unknown	83	13 (16)	5*	38 *
Less than 6 months	5636	389 (7)	90	23 †
6 months to 1 year	3398	350 (10)	75	21
1 year	4282	467 (11)	75	16 †
2 years	1808	203 (11)	34	17
3 years	1066	141 (13)	19	14 †
4 years	671	88 (13)	8	9 †
5 to 9 years	1386	153 (11)	19	12 †
10 to 19 years	1167	58 (5)	8	14
20 to 29 years	1026	43 (4)	4	9
30 to 39 years	1091	82 (8)	5	6 †
40 to 49 years	999	105 (11)	8	8 †
50 to 59 years	1224	116 (10)	8	7 †
60 to 69 years	996	110 (11)	13	12 †
70 to 79 years	595	102 (17)	0	0 †
80 to 89 years	562	104 (19)	2	2 †
90 to 105 years	236	37 (16)	0	0 †

* 13 specimens with unknown age group, of which five had mixed hMPV infection, were excluded from the binary logistic regression analysis. † Using less than six months old as the reference age group, p<0.05 comparing mono versus mixed infection for hMPV among the age groups (Binary Logistics Regression).

**Table 2:** Number and proportion of positive and mixed infection for the nine respiratory viruses and the frequency of the eight viruses identified in specimens with mixed infection with hMPV.

Virus	Number of specimens tested positive by virus	Number of specimens with mixed infection by virus	Number of specimens found in mixed infections with hMPV	% of virus found in mixed infections with hMPV
	(Total number of specimens tested = 26,226*)	(%)	(n=373)	(n=373)
Enterovirus/Rhinovirus	14322	1811 (13)	230 †	56
Respiratory Syncytial virus	5959	1020 (17)	48 †	12
Parainfluenza virus	3296	656 (20)	47 †	11
Human Metapneumovirus	2561	373 (15)	Not applicable	Not applicable
Adenovirus	1317	606 (46)	35 †	9
Coronavirus NL63	381	105 (28)	29 †	7
Coronavirus 229E	349	110 (32)	15 †	4
Coronavirus OC43	293	67 (23)	7 †	2
Coronavirus HKU1	263	82 (31)	2 †	1

* Influenza A and B positive samples and samples not tested by RVP were excluded from the analysis † Some specimens in these subsets had more than one virus identified as co-existing with hMPV

**Table 3:** Age distribution of respiratory virus found in mixed virus infection with hMPV.

		Distribution of virus found in specimens with mixed hMPV infections							
Age groups	Number of specimens with mixed infection with hMPV(n=368*)	ERV	RSV	PIV	ADV	CRV HKU1	CRV 229E	CRV OC43	CRV NL63
Less than 6 month	90 †	60	16	6	8	9	2	0	0
6 month to 1 year	75 †	46	7	6	9	10	2	1	0
1 year	75 †	52	10	9	8	4	1	1	1
2 years	34 †	16	5	6	7	1	2	0	0
3 years	19 †	13	3	3	0	1	0	0	0
4 years	8 †	6	0	2	1	0	0	0	1
5 to 9 years	19 †	9	0	8	0	1	1	1	0
10 to 19 years	8 †	3	2	1	0	1	2	0	0
20 to 29 years	4	4	0	0	0	0	0	0	0
30 to 39 years	5	2	0	2	1	0	0	0	0
40 to 49 years	8	3	2	1	0	1	1	0	0
50 to 59 years	8	2	1	1	0	1	3	0	0
60 to 69 years	13 †	9	1	1	0	0	1	4	0
70 to 79 years	0	NA	NA	NA	NA	NA	NA	NA	NA
80 to 89 years	2	1	0	1	0	0	0	0	0
90 to 105 years	0	NA	NA	NA	NA	NA	NA	NA	NA

* Five specimens with unknown age group were not tabulated † Some specimens in these subsets had more than one virus identified as co-existing with hMPV

The objective of this study was to describe mono and mixed infections of hMPV in our jurisdiction for a three year period. All specimen data was acquired using ProvLab’s DIAL application and we were able to identify that hMPV was more likely to occur in mono infection (85%) than mixed (15%). The results in this study were similar to a previous pilot study in Alberta which identified hMPV mixing with other pathogens in 15% of specimens (118/778) [17]. In other populations, namely hospitalized patients, mixed hMPV infections were in the minority.

Our study also determined that in hMPV mixed infections, the pathogen most likely to co-exist with hMPV is ERV (56%). In contrast, other studies have identified RSV as the leading cause of mixed infection with hMPV [18,19,20]. There may be several reasons for the difference in findings including the type of diagnostic assays used and the detection of various respiratory targets, clinical setting, patient age, and timing of specimen collection. Many studies have focused on populations mostly consisting of hospitalized pediatric patients and these studies have shown that RSV was the leading pathogen identified in these young children who were between 16 months to 14 years old [21,22,23]. In contrast, our study examined all age groups, including community-based and hospital-based specimens. ERV and hMPV remained closely linked across all age groups. Mixed infection with hMPV was most commonly found in the age group less than 6 months old. Moreover, ERV was still the dominant virus found to be mixed with hMPV. Our data set included specimens collected during the second phase of 2009 H1N1 pandemic in Alberta and Northern Territories, which made up of more than one third of the specimens included in this study. We found 62% of the samples between July 2009 and June 2010 tested positive for ERV, significantly higher than that found in the other two time periods, July 2010 to June 2011 (49%) and July 2011 to June 2012 (51%) (p<0.001, Binary Logistic Regression). Other studies have also shown that during the pandemic ERV was a very common circulating virus followed by CRV [22,24,25]. Moreover, this linkage between ERV and hMPV could also be due to the inclusion of a very large group of viruses identified as ERV since RVP could not distinguish between various strains of rhinovirus and enterovirus. There might also be factors related to host immune responses and viral infection kinetics [26,27,28,29]. We have only examined the seasonality of hMPV and ERV and the mixed infection between these two viruses because of the relatively lower number of hMPV mixed infections for the other six viruses. Annual variations in peak hMPV activity was observed in our study with one of the peak months occurring in the summer of 2011, which was different from other studies in various Canadian provinces showing peaks of hMPV in the winter (January to April) [30,31,32]. As expected, the mixed infection of hMPV and ERV followed the circulatory pattern of hMPV but differed from ERV.

Although our findings have shown some trends with hMPV and its ability to exist as mono versus mixed infection, there are some important limitations in this study. Firstly, influenza A and B positive samples were excluded mainly because ProvLab does not routinely test influenza positive specimens for hMPV since the pandemic 2009. Excluding influenza viruses from our database may have impacted viral co-infection rates identified in our study. On the other hand, a recent study in England showed a lower prevalence of influenza A (H1N1) in patients positive for hMPV, ADV, RSV, PIV and rhinovirus with statistical significance for this relationship with hMPV and rhinovirus [32]. Another limitation was that the analysis by age group was based on specimen-based data only with duplicate samples from some individuals. Moreover, only limited information is usually provided on the requisition submitted with the specimens so we were not able to study clinical manifestations of mono-infection versus mixed infections and explore different settings

Despite the limitations stated, this study has considerable public health implications. This study helps us better understand the frequency of hMPV mono versus mixed infection over a period of three years. Better understanding of what mechanisms or conditions support mono versus mixed infections and the difference in clinical presentations and prognosis is needed. The prevalence of this virus supports research efforts to develop vaccine strategies which may become available in the future [33]. This also brings us to question whether testing algorithms at ProvLab need to be changed to better understand the interaction between hMPV and influenza A and B. Since hMPV has been shown to be an important pathogen, it should be included in ongoing surveillance and public health strategies. Enhanced surveillance programs will help us better understand hMPV-associated diseases and maintain our awareness of trends in mono and mixed infections.

## 3. Experimental Section

ProvLab provides respiratory virus testing for the province of Alberta and surrounding Northern Territories (excluding Yukon) of Canada. Since June 2009, all respiratory specimens arriving at ProvLab was screened by an in-house (RT)-PCR for influenza A [14]. Specimens tested positive for influenza A were also subtyped for seasonal H3, seasonal H1 and pandemic H1N1 (2009) genes by RT-PCR. A multiplex assay to detect both influenza A and B was implemented in February 2010. Only specimens tested negative for both influenza A and B were tested using the RVP classic assay, a multiplexed assay which detects multiple respiratory viral pathogens including FLUA, FLUB, PIV, ERV, ADV, four types of CRV, RSV, and hMPV [14]. Exceptions to this testing policy included samples submitted from a provincial influenza-like-illness surveillance program (Tarrant Viral Watch) and some samples from patients with severe illness and admitted to the intensive care units. Specimens tested by RVP and tested positive for one or more respiratory virus excluding influenza A and B between July 2009 and June 2012 identified using the DIAL application was included in this study. DIAL provided classification of the specimens by each respiratory virus target as well as mono versus mixed infection. DIAL also allowed a user to select specimens based on the type of testing and over various geographic and time periods and age groups.

Statistical analysis of the proportion of mono versus mixed infections for the different virus was performed using Pearson Chi-squared (χ^2^) test with Bonferroni’s adjustment for multiple analyses. The proportion of hMPV as mixed infection by age group and ERV positive specimens by analysis of age group and time periods was analyzed using binary logistic regression. The SPSS software version 20.0 (IBM® SPSS® Statistics, IBM Corp., USA) was used for statistical analysis with the level of significance set at p<0.05. 

## 4. Conclusions

In this study we used DIAL to provide user-defined data sets of respiratory virus testing data and found that over a period of 3 years, July 2009 to June 2012, hMPV is significantly more likely to be identified in mono infection (86%) as compared with ADV, four types of CRV, PIV, and RSV with the exception of ERV. The three viruses most likely to be found in mixed infection with hMPV in this study were ERV, PIV and RSV with a higher proportion of mixed infection found in young infants.

## References

[1] Statistics Canada Leading causes of death in Canada 2009. http://www.statcan.gc.ca/pub/84-215-x/2012001/tbl/T001-eng.pdf.

[2] van den Hoogen B.G., de Jong J.C., Groen J., Kuiken T., Fouchier R.A., Osterhaus A.D. (2001). A newly discovered human pneumovirus isolated from young children with respiratory tract disease. Nat. Med..

[3] Liu L., Bastien N., Sidaway F., Chan E., Li Y. (2007). Seroprevalence of human metapneumovirus (hMPV) in the Canadian province of Saskatchewan analyzed by a recombinant nucleocapsid protein-based enzyme-linked immunosorbent assay. J. Med. Virol..

[4] Manoha C., Espinosa S., Aho S.L., Huet F., Pothier P. (2007). Epidemiological and clinical features of hMPV, RSV and RVs infections in young children. J. Clin. Virol..

[5] Mullins J.A., Erdman D.D., Weinberg G.A., Edwards K., Hall C.B., Walker F.J., Iwane M., Anderson L.J. (2004). Human metapneumovirus infection among children hospitalized with acute respiratory illness. Emerg. Infect. Dis..

[6] Kaida A., Iritani N., Kubo H., Shiomi M., Kohdera U., Murakami T. (2006). Seasonal distribution and phylogenetic analysis of human metapneumovirus among children in Osaka City, Japan. J. Clin. Virol..

[7] Falsey A.R., Erdman D., Anderson L.J., Walsh E.E. (2003). Human metapneumovirus infections in young and elderly adults. J. Infect. Dis..

[8] Boivin G., Abed Y., Pelletier G., Ruel L., Moisan D., Cote S., Peret T.C., Erdman D.D., Anderson L.J. (2002). Virological features and clinical manifestations associated with human metapneumovirus: a new paramyxovirus responsible for acute respiratory-tract infections in all age groups. J. Infect. Dis..

[9] Boivin G., De S.G., Hamelin M.E., Cote S., Argouin M., Tremblay G., Maranda-Aubut R., Sauvageau C., Ouakki M., Boulianne N., Couture C. (2007). An outbreak of severe respiratory tract infection due to human metapneumovirus in a long-term care facility. Clin. Infect. Dis..

[10] Louie J.K., Schnurr D.P., Pan C.Y., Kiang D., Carter C., Tougaw S., Ventura J., Norman A., Belmusto V., Rosenberg J., Trochet G. (2007). A summer outbreak of human metapneumovirus infection in a long-term-care facility. J. Infect. Dis..

[11] Towgood L., Miller M., McKay D., Parker R. (2008). Human metapneumovirus outbreak in a senior's care facility, British Columbia. Canada Communicable Disease Report (CCDR) Weekly.

[12] van Asten L., van den Wijngaard C., van Pelt W., van de Kassteele J., Meijer A., van der Hoek W., Kretzschmar M., Koopmans M. (2012). Mortality attributable to 9 common infections: significant effect of influenza a, respiratory syncytial virus, influenza B, norovirus, and parainfluenza in elderly persons. J. Infect. Dis..

[13] Xie Z.D., Xiao Y., Liu C.Y., Hu Y.H., Yao Y., Yang Y., Qian S.Y., Geng R., Wang J.W., Shen K.L. (2011). Three years surveillance of viral etiology of acute lower respiratory tract infection in children from 2007 to 2010. Zhonghua Er. Ke. Za Zhi..

[14] Pabbaraju K., Tokaryk K.L., Wong S., Fox J.D. (2008). Comparison of the Luminex xTAG respiratory viral panel with in-house nucleic acid amplification tests for diagnosis of respiratory virus infections. J. Clin. Microbiol..

[15] Lee B.E., Mukhi S.N., May-Hadford J., Plitt S., Louie M., Drews S.J. (2011). Determination of the relative economic impact of different molecular-based laboratory algorithms for respiratory viral pathogen detection,including Pandemic (H1N1),using a secure web based platform. Virol. J..

[16] Mukhi S.N., May-Hadford J., Plitt S., Preiksaitis J., Lee B.E. (2012). DIAL: A Platform for real-time Laboratory Surveillance. Online Journal of Public Health Informatics.

[17] Pabbaraju K., Wong S., McMillan T., Lee B.E., Fox J.D. (2007). Diagnosis and epidemiological studies of human metapneumovirus using real-time PCR. J. Clin. Virol..

[18] Boivin G., De S.G., Cote S., Gilca R., Abed Y., Rochette L., Bergeron M.G., Dery P. (2003). Human metapneumovirus infections in hospitalized children. Emerg. Infect. Dis..

[19] Kuypers J., Wright N., Corey L., Morrow R. (2005). Detection and quantification of human metapneumovirus in pediatric specimens by real-time RT-PCR. J. Clin. Virol..

[20] Wolf D.G., Greenberg D., Kalkstein D., Shemer-Avni Y., Givon-Lavi N., Saleh N., Goldberg M.D., Dagan R. (2006). Comparison of human metapneumovirus, respiratory syncytial virus and influenza A virus lower respiratory tract infections in hospitalized young children. Pediatr. Infect. Dis. J..

[21] Sung C.C., Chi H., Chiu N.C., Huang D.T., Weng L.C., Wang N.Y., Huang F.Y. (2011). Viral etiology of acute lower respiratory tract infections in hospitalized young children in Northern Taiwan. J. Microbiol. Immunol. Infect..

[22] Xiao N.G., Xie Z.P., Zhang B., Yuan X.H., Song J.R., Gao H.C., Zhang R.F., Hou Y.D., Duan Z.J. (2010). Prevalence and clinical and molecular characterization of human metapneumovirus in children with acute respiratory infection in China. Pediatr. Infect. Dis. J..

[23] Martin E.T., Kuypers J., Wald A., Englund J.A. (2012). Multiple versus single virus respiratory infections: viral load and clinical disease severity in hospitalized children. Influenza. Other Respi. Viruses.

[24] Esper F.P., Spahlinger T., Zhou L. (2011). Rate and influence of respiratory virus co-infection on pandemic (H1N1) influenza disease. J. Infect..

[25] Thiberville S.D., Ninove L., Vu Hai V, Botelho-Nevers E., Gazin C., Thirion L., Salez N., de Lamballerie X., Charrel R., Brouqui P. (2012). The viral etiology of an influenza-like illness during the 2009 pandemic. J. Med. Virol..

[26] Bitko V., Shulyayeva O., Mazumder B., Musiyenko A., Ramaswamy M., Look D.C., Barik S. (2007). Nonstructural proteins of respiratory syncytial virus suppress premature apoptosis by an NF-kappaB-dependent, interferon-independent mechanism and facilitate virus growth. J. Virol..

[27] Spann K.M., Tran K.C., Chi B., Rabin R.L., Collins P.L. (2004). Suppression of the induction of alpha, beta, and lambda interferons by the NS1 and NS2 proteins of human respiratory syncytial virus in human epithelial cells and macrophages [corrected]. J. Virol..

[28] Ditt V., Lusebrink J., Tillmann R.L., Schildgen V., Schildgen O. (2011). Respiratory infections by HMPV and RSV are clinically indistinguishable but induce different host response in aged individuals. PLoS One.

[29] Franz A., Adams O., Willems R., Bonzel L., Neuhausen N., Schweizer-Krantz S., Ruggeberg J.U., Willers R., Henrich B., Schroten H., Tenenbaum T. (2010). Correlation of viral load of respiratory pathogens and co-infections with disease severity in children hospitalized for lower respiratory tract infection. J. Clin. Virol..

[30] Hamelin M.E., Abed Y., Boivin G. (2004). Human metapneumovirus: a new player among respiratory viruses. Clin. Infect. Dis..

[31] Robinson J.L., Lee B.E., Bastien N., Li Y. (2005). Seasonality and clinical features of human metapneumovirus infection in children in Northern Alberta. J. Med. Virol..

[32] Bastien N., Ward D., Van C.P., Brandt K., Lee S.H., McNabb G., Klisko B., Chan E., Li Y. (2003). Human metapneumovirus infection in the Canadian population. J. Clin. Microbiol..

[33] Kahn J.S. (2007). Newly discovered respiratory viruses: significance and implications. Curr Opin Pharmacol..

